# Tinea Kerion

**Published:** 1879-11

**Authors:** Wm. H. Fitch

**Affiliations:** Rockford, Ill.


ARTICLE IX.

*I. Tinea Kerion.*

Master Charley S., aged 9, came from the country to the office March 31st, 1879, presenting, on the crown of the head, an oblong tumefaction two by three inches. This had been first noticed three weeks previously and treated by domestic and other remedies, without in any way apparently interfering with its progress. It was crusted over and gave such undoubted signs of fluctuation, as these cases often do, that I opened it, but instead of a free discharge, there could only be pressed out a muco-purulent gummy substance. The swelling was quite tender, as well as the scalp immediately surrounding it. There had been some tenderness of the scalp noticed for some time before the disease was discovered. He complained of loss of appetite and of feeling tired. The patient presented several other spots of the disease as large as a finger-nail on other parts of the head, that resembled impetigo. The parents were directed to shingle the the boy's head. Apply olive oil freely at night and a poultice during the day, and return in two days.

April 2d.—Removed the poultice, and with it came away the crust and all the hair from the diseased spots, revealing a raw boggy honey-combed surface, exuding a muco-purulent, gummy substance, varying in color from chocolate to yellow. All the hairs were removed bordering on the diseased spots and epilating forceps were given the parents to continue the process on all loose hairs. The following lotion was ordered to be applied several times a day:

| | | |
|---|---|---|
| ℞ Hydrarg bichlorid | | 12 |
| Sodii hyposulphitis | 8 | |
| Aq. rosæ | 128 | |

M.

Also to take internally 10 minims of syrup of iodide of iron after each meal, in water.

April 5th.—But slight discharge from sore. Swelling much diminished. Healing at circumference. Soreness mostly gone. The smaller spots entirely healed. General health and appetite much better. Same treatment continued.

April 9th.—Swelling entirely gone. Scarcely any discharge. Boy complains of spot itching. Same treatment continued.

April 30th.—Patient cured. Surface smooth and shiny with soft, fine hairs returning around the borders. Ordered a stimulating lotion of

| | |
|---|---|
| Tr. cantharides | 8 |
| Tr. nuc. vom | 10 |
| Tr. capsici | 4 |
| Glycerine | 32 |
| Aq. cologn | 74 |

June 30th.—Hair growing nicely over entire surface.

Microscopical appearance of diseased hairs.

Kerion is simply an aggravated form of tinea tonsuranus or ring-worm of the scalp, due to the presence of the trichophyton. A microscopical examination of the hairs shows them to be permeated with spores and mycelium, even as in these we examined, to such a degree as to rupture the hair. Where sufficient inflammation exists at the roots of the hairs to produce suppuration the disease resembles sycosis.

Two or three other children in the neighborhood of my patient were similarly afflicted, as nearly as I could learn. Some of them were cured by borrowing my patient's bottle, and the others by the application of lime water. As they all attended the same district school, they probably acquired the disease by exchange of caps.

*II. Pustular Eczema of Scalp.*

Mrs. C., aged 26, sent for me, June 15, to see her, on account of a disease of her scalp which some one had called erysipelas, but as it did not exactly correspond to their notions of this disease, I was called. Mrs. C. is thin and tall; had always enjoyed good health until recently, when she has been suffering from general debility. She never had had any skin disease except eruptive fevers. Her mother had suffered from varicose ulcers a year or two previously, which healed readily under the solid rubber bandage. The present attack was preceded by chill, headache and bone-ache. There first appeared, a few days previous to my visit, a crop of pustules in the occipital region, accompanied by much tenderness, heat and pain, with enlargement and soreness of post-cervical glands, some of which were suppurating. There were also two patches in parietal region on either side of median line, each about 7½ ctm. long by 2½ ctm. broad, considerably elevated above the surrounding surface, presenting a suppurating, uneven, infiltrated, honey-combed appearance, much resembling the kerion in the previous case. The hairs were loose, and had mostly fallen out from these surfaces, and with the previous case in mind, I prescribed a parasiticide lotion, and brought away a number of hairs for microscopic examination, which, however, only yielded negative results. The whole scalp was so tender that the patient was unable to lay her head in any position for sleep, which she obtained only by considerable doses of anodynes.

June 18th. — Patient no better. Swelling or œdema extends to forehead and face, nearly closing the eyes. The diseased patches were covered with crusts and matted hair. The eruption had become so universal that it seemed best to cut the hair short. Ordered to have the head saturated with almond oil over night, and thoroughly washed out in the morning with soap and water, which was repeated several nights; during the day the scalp to be frequently wet with blackwash.

Internally she was ordered a desert-spoonful of elixir calasaya, iron and bismuth 3 times a day, and effervescing salines.

June 22d.—Less swelling about face and scalp, but soreness largely remains. The larger patches appear somewhat better.

Ordered an ointment of calomel 4 grams, vaseline 32 grams, to be applied twice a day. Internally, ammonia, ferri citrat. and strychnia.

June 28.—Patient has a good appetite and sleeps well, but some new patches of pustules have appeared and the older ones are most inclined to heal satisfactorily.

Ordered ol. oliv., Goulard's ext. āā grams 32, tr. arnica grams 16. To be applied twice a day.

Internally,

| | | | |
|---|---|---|---|
| Acid arsenoisi | | | |
| Strychnia sulph | āā | | 06 |
| Tr. ferri | | | |
| Acid hydrochloric dil. | āā | 16 | |
| Cinchonidæ sulph | | 2 | |
| Elixir simpl., ad | | 128 | |

Dose a teaspoonful after meals in a wine glass of water.

July 1st.—Patient presented herself at office nearly cured. Said the local application stopped the discharge almost immediately, and with it the pain itching and soreness ceased.

July 10.—Patient well, hair returning on diseased patches, and no new pustules have appeared. I have reported this case simply on account of the liability there is of confounding it with kerion, herpes and favus.

WM. H. FITCH.

ROCKFORD, Ill.

